# Why a Virtual Assistant for Moral Enhancement When We Could have a Socrates?

**DOI:** 10.1007/s11948-021-00318-5

**Published:** 2021-06-29

**Authors:** Francisco Lara

**Affiliations:** University of Granada, Granada, Spain

**Keywords:** Moral enhancement, Moral bioenhancement, Moral AIenhancement, Artificial intelligence, Virtual assistant, Ethical decision-making, Autonomy

## Abstract

Can Artificial Intelligence (AI) be more effective than human instruction for the moral enhancement of people? The author argues that it only would be if the use of this technology were aimed at increasing the individual's capacity to reflectively decide for themselves, rather than at directly influencing behaviour. To support this, it is shown how a disregard for personal autonomy, in particular, invalidates the main proposals for applying new technologies, both biomedical and AI-based, to moral enhancement. As an alternative to these proposals, this article proposes a virtual assistant that, through dialogue, neutrality and virtual reality technologies, can teach users to make better moral decisions on their own. The author concludes that, as long as certain precautions are taken in its design, such an assistant could do this better than a human instructor adopting the same educational methodology.

## Introduction

The latest technological advances could substantially change our way of being. It is a matter of debate in contemporary applied ethics whether or not to use these advances to enhance ourselves as moral agents. In this article, I will argue that, provided some conditions are met, the use of Artificial Intelligence (AI) could be the most appropriate in this regard.

To do so, I will devote the first section to analysing the current proposals for “moral enhancement” of human beings through two different technologies in order to show, in my opinion, which path should not be taken in this matter. I will begin with the proposals that draw upon the use of the latest advances in neuroscience, and specifically biotechnology, which I will include under the term “moral bioenhancement”. I will argue that they do not achieve their objective because they neglect the notion that an individual’s morality cannot be strengthened without taking their autonomy seriously. In the rest of the section, I will attempt to prove that it is precisely this same error that also invalidates the positions of those who have hitherto defended that what we need to morally enhance human beings is not biotechnology but rather another new field of technological expansion: AI. These positions are encompassed under the neologism “moral AIenhancement”.

The conclusions of the first section will serve to devise, in the second section, a virtual assistant that, through various AI-related technologies, truly strengthens our morality thanks to its effectiveness in fostering personal autonomy. I will argue that the assistant should seek the maximum deliberative preparation of the user through a dialogue of clear Socratic inspiration, but must be governed by procedural ethical criteria that ensure axiological neutrality.

Even so, it is worth challenging this proposal as to whether we need a virtual assistant for something that could be achieved, to an equal or greater extent, with an ethical human instructor implementing a similar method. In the final section, I will therefore perform a comparison between the proposed virtual assistant and its human counterpart. The comparison will lead me to conclude that, as long as certain precautions are taken, the virtual option is preferable.

## No Enhancement Without Autonomy

### The Passivity of the Bioenhancement Process

The ethical debate on moral enhancement dates back to the beginning of the century and has focused, most of the time, on moral bioenhancement (Agar, 2010, 2015; Douglas, 2008; Faust, 2008; Harris, 2016; Persson & Savulescu, 2008). Most authors in support of this largely believe that any biological intervention that strengthens certain moral emotions or motivations, such as altruism, or reduces other immoral ones, such as aggression, represents an enhancement in itself (Persson & Savulescu, 2008, 2012; Douglas, 2008, 2013; Crockett, 2014; Earp et al. 2018)^1^ However, this is a misguided approach since it fails to realise that the price to pay for these kinds of interventions is usually the erosion of personal autonomy. Autonomy must be safeguarded because it is a crucial factor for well-being and the definition of what a person is. This is why some authors believe that the promotion of this capacity is the foundation of political and moral systems (Mill, 1859/1975: c. 3, Sen, 2010, p. 18). This being so, it would be inaccurate to claim that a biological intervention that reduces autonomy can lead to a "moral enhancement" of people.

But in what sense can it be argued that such interventions erode autonomy? This could be argued in two ways. The first would be to point out that if by changing the person's biology we increase his willingness to do the right thing, we are considerably reducing his behavioural options. We would be depriving him of the possibility of doing the wrong thing, of the "freedom to fall" (Harris, 2011). But, as some authors have argued, not having action alternatives to choose from does not make one less free (Savulescu & Persson, 2012, p. 409; Douglas, 2013; DeGrazia, 2014, pp. 5–7). To see this, Savulescu and Persson (2012) ask us to imagine an intelligent computer, the "God Machine", which allows people to act freely as long as this does not entail great harm or injustice. They argue that, even if in such a hypothetical situation, people's real choices were fewer, they would still be acting freely. This would explain our usual belief that people who, because of their moral zeal, consider no alternative but to do the right thing, are no less free than immoral people (Persson & Savulescu, 2013, p. 128). Therefore, what moral enhancement, be it biotechnological or educational, would achieve in this case is not a limiting of autonomy, but the possibility “to make the unacceptable unpalatable, not undoable” (Harris, 2013, p. 170, in a different context).

So, when I argue that bioenhancement entails an erosion of personal autonomy, I mean it in a second sense. I do not wish to refer to autonomy as the possibility of acting otherwise (of choosing the "moral fall"), but as self-determination, that is, relating it more directly to the will of the individual than to its results (on the distinction, see Ekstrom, 2012). In this other sense, actions are autonomous when they are governed by the individual, not by an external will. This means two things. First of all, that the individual must identify with the values underlying the action, and with the judgments derived from them, after having subjected them to some rational consideration. The individual must be able to make sense of his life from a higher perspective that reflects on his acquired ("first-order") preferences, desires, values, etc. (Dworkin, 1988, p. 25; 1989; Frankfurt, 1971; Arneson, 1991). But, secondly, autonomy as self-determination also means that the individual must have sufficient self-confidence, resolve and self-control to act in accordance with these values and judgments (Berofsky, 1995; Dworkin, 1976; Haworth, 1986). As recent empirical studies show (Moll et al., 2005; Wiseman, 2016; Casebeer and Churland 2003; Decety & Howard, 2013, pp. 49, 53; Pascual et al. 2013; Young & Dungan, 2012), autonomous moral decisions always involve an interaction of the affective, the cognitive and the motivational. We can say that a person is involved with his or her values and that he or she considers them truly his or her own when, to the extent of his or her possibilities, he or she is willing to behave by them. In this sense, we can say that an individual chooses their values autonomously when they are the result of a balance between capacities or attitudes of both an affective and rational nature reached personally by the individual. It is a balance that continues to exist whenever the person also uses these capacities and attitudes to make decisions, to flexibly deliberate on what to do in a given situation (Schaefer, 2015; Earp et al. 2018, Carter & Gordon, 2015). It is an internal balancing act which, despite occasionally being facilitated by experience or by following simple, useful and justified guidelines for action, reaches a high degree of difficulty in morally dilemmatic or novel situations.

In what way, then, could the change in moral emotions sought by the advocates of bio-enhancement negatively affect this capacity for self-determination? In a first sense, it would do so if the change were made against the manifest will of the subject. Even if it were for its own sake, to impose it coercively would constitute an inadmissible paternalism in the sense, defended by Dworkin (1972, p. 83), of externally interfering in the values that are decisive in the life of the individual. The individual would be drastically stripped of something as important as having a say in the constitution or modification of his or her own identity (Klincewicz 2016, p. 183). But what I am arguing here is that, with such interventions, this 'silencing' would still be present, in a way, even when they are carried out with the individual's consent since the two basic elements of self-determination would be undermined in such cases. Firstly, by aiming primarily at influencing attitudes, the techniques are used in a way that bypasses reasoning (Harris, 2014, p. 372), which leads to giving the subject in the process the role of a mere "passive recipient" (Schaefer, 2015, p. 268; Raus et al., 2014; Schermer, 2015). This is what makes it crucially different from other traditional forms of moral 'enhancement', such as cognitive therapy or education at advanced ages, where significant involvement and effort is usually required from the individuals to enhance (Focquaert & Schermer, 2015). In these other participatory interventions, there is room for the subject to progressively deliberate on the changes taking place within him/her and, if he/she does not identify with them, to withdraw from the intervention or to select which changes to accept. This is not the case, however, when the intervention involves a direct alteration of the nervous system in order, for example, to make the individual more altruistic.

But it is not only a question of these interventions limiting the deliberative capacity of the subject. They also negatively affect that second element of autonomy having to do with authenticity. While it is true that beliefs, desires and personality traits are dynamic, changes in these psychological factors must be incorporated into one's 'life story' in a coherent way, and without compromising the sense of self. This is similar to what Dworkin (1972) demanded of methods of social influence: that, in addition to not dispensing with the participation of individuals, they should not cause sudden discontinuities in their unified conception of themselves. It is precisely this narrative identity that could be abruptly affected by the immediate changes of bio-enhancement (Focquaert & Schermer, 2015, pp. 145–146; Schechtman, 2010). Some studies show that patients who undergo deep brain stimulation find it difficult to come to terms with the psychological and functional changes that this technique brings about and to adopt a new self-image (Gisquet, 2008). In some cases, they become depressed because they feel alienated or confused by their new identities (Schechtman, 2010, p. 137).

There are two possible responses to my criticism of moral bioenhancement for its excessive conditioning of the subject. The first would be to argue that, in certain situations and if conducted properly, intervention in moral emotions could be compatible with the active participation of the subject and, therefore, not erode autonomy; it may even increase it. Savulescu and Persson (2012, pp. 411–412) propose the hypothetical case of a pill that "clarifies the view" that a usually selfish person has of the other, thus allowing his or her moral deliberation to be more complete and motivating. Even so, they add, decision-making would still require effort and learning. Other authors make similar arguments concerning bioenhancement as increasing "moral impulse control" (Earp et al., 2015) or neutralising counter-moral emotions (Douglas, 2008), presenting it, therefore, as liberating us from that which prevents us from being truly autonomous. The problem with these bioenhancement proposals is that, given the foreseeable neurological advances, they cannot be implemented in the near future, as some of their advocates acknowledge (Douglas, 2008, p. 166). To really increase moral attitudes without simultaneously undermining autonomy would require a difficult "fine-tuning" of emotions that would be sensitive to the varied particularity of individuals and the circumstances they may face. Without such a technical possibility, moral bioenhancement could be counterproductive, depriving individuals, for example, of the ability to express the right, sometimes necessarily aggressive, reactions to grave injustices (Chan & Harris, 2011, p. 131; Harris, 2011, p. 105; Dees, 2011, p. 13).

The second possible response to the above critique of moral bioenhancement would be, while acknowledging the threat to personal autonomy, to maintain that this could be compensated for by the increased well-being and quality of life for many beings that the increased moral motivation of the enhanced people would bring (DeGrazia, 2014; Savulescu & Persson, 2012, p. 416). It could even be added that such gains could justify that sometimes, as with medical interventions, enhancement is carried out at the risk of possible accidents, cognitive limitations or unintended negative effects (Douglas, 2013).

I will not assess here the acceptability of this possible counterargument, but I will consider it as a point of reference to ask, in the rest of the article, whether such positive achievements of moral enhancement could be achieved with AI in a better way, i.e. without diminishing personal autonomy and without the risks currently associated with the use of biotechnology for this purpose.

### Ethics Machines, Nudges and Ethical Advisors

Moral AIenhancement is a good alternative because, by not aiming to directly change motivational aspects of behaviour, it would, in principle, pose less risk to autonomy. Let's see how well this expectation is fulfilled in each of the three models I envision in the emerging debate on moral AIenhancement.

The first model would consist of an extrapolation to this debate of some achievements in what is known as “machine ethics”. The objective of this field is to contribute to the configuration of autonomous and robotic machines so that they can function by themselves in morally difficult situations following ethical criteria. For example, system designers might equip driverless vehicles with algorithms allowing them to choose between the different victims of their possible reactions in unavoidable accidents. Some authors suggest that we could use these kinds of advances in machine ethics to design “ethics machines”, systems that direct the behaviour of human beings, either by replacing them completely when making decisions (Dietrich, 2001), or by overriding or correcting them (Gips, 1995). They justify the heavy dependence on machines that this would entail for humans by the supposedly unwavering impartiality, consistency and equanimity of the former, and the egoism, deliberative fatigue and group favouritism characteristic of the latter.^2^

Whether or not this justification of the model is valid, there are reasons to doubt its viability. But what interests me here is to highlight another major deficiency of the model: its negative impact on personal autonomy. If to do the right thing, we need only obey a machine whose ethical algorithms are determined from the perspective of the designer, our role will always be largely passive and the reasons to behave morally will always come from the outside ((Lara & Deckers, 2020, pp. 277, 279–280).

We could then consider a second moral AIenhancement model in which, in order to protect the autonomy of the user, the recommendations of the machine could be rejected at any time. To do so, we could make use of nudges, widely discussed since being popularised by Sunstein and Thaler (2003; Thaler & Sunstein, 2008), especially in the commercial and public health fields. A nudge is any aspect of a choice architecture, or decision-making environment, that aims to influence people to supposedly make better decisions for their welfare whilst always leaving their freedom of choice intact, i.e., not prohibiting particular choices or significantly changing incentives (Thaler & Sunstein, 2008; Sunstein, 2015a, pp. 7–8). The vagueness of this definition, however, means that nudges can encompass a wide variety of interventions. For example, strategies that aim to influence behaviour by simply providing information, such as making apples more visible than unhealthy products in a cafeteria (Thaler & Sunstein, 2008) or displaying a certain route on a GPS device, would be nudges. In such cases, highlighting or simply providing certain information can be considered a nudge because, by virtue of the fact that such information usually elicits a similar reaction from people, it is expected to alter behaviour in a predictable way. But there are also more sophisticated nudges that draw on a deeper knowledge of human behaviour, particularly with regard to certain decision-making heuristics or biases that we often utilise in order to make decisions quickly and according to certain cognitive cues rather than to all the available information. Some nudges therefore aim to improve the individual's decision-making by getting the individual to block such cognitive shortcuts, warning, for example, of the convenience of undergoing a period of reflection before taking a certain action. Finally, there are the more ethically problematic nudges which aim, by changing the choice architecture, to trigger these shortcuts to steer people's behaviour in specific directions (Barton & Grüne-Yanoff, 2015, p. 343).

Some authors have suggested that nudges could inspire the design of robots that promote the necessary attitudes and skills for humans to behave by following some ethical standards (Borenstein & Arkin, 2016; Klincewicz, 2019). Although these proposals would specifically target robots to take advantage of certain benefits of a humanoid chassis, such as emotive influence (Asada et al., 2009), gesture communication (Brooks & Arkin, 2007) or the inspiration of more authority (Aroyo et al., 2018), they could also be implemented in simple computer programmes (Klincewicz, 2019, pp. 426–427).

The impact of these proposals on user autonomy will depend on the type of nudge they are based on. It will not negatively affect autonomy, and may even improve it, if the proposal lends special importance to those nudges that, ultimately, aim to enhance the user's decision-making by encouraging them to be more reflective in certain situations. Such is the case, for example, of the proposal by Klincewicz (2019), who proposes designing social robots that enhance user morality by inclining them towards strategies in line with the practical advice of the ancient Stoic philosophers. The nudges would serve here to free the user from those emotional blocks that normally hinder our cold and efficient reflection, such as worrying about issues whose resolution is beyond our control, ruminating about past events or not realising the irrationality of many of our emotions (Klincewizc, 2019, pp. 436–439).

Quite different is the case of those proposals that resort to nudges to trigger decision-making heuristics that incline the user's decision to certain substantive ethical stances. An example would be the one suggested, in merely illustrative terms, by Borenstein and Arkin (2016). These would be companion robots designed to, by means of (dis)incentive strategies similar to those that humans use with each other, foster beliefs and attitudes in accordance with Rawls' principles of justice. User autonomy could certainly be thought to be safeguarded in this model because, although the ultimate goal of nudges is to increase the likelihood of one option being chosen, in pushing the individual in one specific direction, they do so with a libertarian paternalism that, while intending the best for the individual, always preserves their freedom to oppose (Thaler & Sunstein, 2008, pp. 4–6). However, the objection can always be raised that this preserved freedom is minimal and insufficient precisely because these types of nudges aim to subtly alter the behaviour of the individual. Instead of influencing through reason and arguments, the heuristics-triggering nudges take advantage of some of the character traits of the individual to achieve an easier adherence to the aims of the designer. In other words, the very tactic that characterises these nudges entails in itself an attempt to circumvent the deliberative capacities of the individual, thus significantly limiting autonomy (Ashcroft, 2013; Bovens, 2009; Hausman & Welch, 2010; MacKay & Robinson, 2016; Saghai, 2013; Wilkinson, 2013; Yeung, 2012). This intended adherence of the individual to the external aims would correspond more to immediate, superficial and *blind* acceptance than to a reflective personal identification with them. Some authors even argue that the threat to autonomy posed by nudges comes from their supposedly manipulative nature. Manipulation occurs when one influences another by bypassing their capacity for reason, either by taking advantage of the non-rational elements of their psychology or by influencing their decisions in a non-transparent way that is not obvious to the subject. This is precisely what happens, these authors point out, in certain nudges (Blumenthal-Barby & Burroughs, 2012, p. 5; Hausman & Welch, 2010, p. 136; Grüne-Yanoff, 2012, pp. 636–637).

In the case of technological nudges, such as those that could be used for this model of moral AIenhancement, the threat to autonomy would be even greater. In contrast to other types of influences, the nudging that would be possible through AI assistants, whether robotic or not, would benefit from the technology's own ability to find useful correlations between data "not capable of analysis by ordinary human assessment" (Shaw, 2014). It would be a kind of “hypernudging”, especially subtle, unobtrusive and tremendously powerful. By learning from the user's past behaviours and preferences, the assistant could constantly and dynamically update the choice architecture in a way that would make preferable behavioural options more appealing (Yeung, 2017). Moreover, this hypernudging of assistants would make it impossible to fulfil that condition of publicity and transparency that, for some authors, would render nudges non-manipulative, making them "visible, scrutinized and monitored" (Sunstein, 2015b, pp. 147–148). The assistants would be designed with influence mechanisms based on complex machine learning algorithms, which would make them highly opaque (Yeung, 2017, p. 124).

It can therefore be concluded that autonomy may not be sufficiently respected in either of the models presented because, in one way or another (replacing or pushing in one direction), the subject's values are not the determining factor. This could be avoided if the computer programme were designed with the sole intention of assisting the user in moral decision-making. The result would be an ethical advisor that would provide the user with guidelines which, in addition to being subject to rejection or revision at any time, would be based on the user's own moral values. This would be the underlying idea for what we could consider a third moral AIenhancement model and which has been laid out in two proposals, Savulescu and Maslen (2015), on the one hand, and Giubilini and Savulescu (2017), on the other. In the first, the user would choose and organise, by virtue of their priorities, the basic values from a list provided by the system. The advisor would then process the information at its disposal according to this hierarchy of values and recommend guidelines of moral behaviour to the user. In the second proposal of this model, Giubilini and Savulescu, the user must choose a version of the advisor system that fits their personal values. Then, from the version chosen, the system would suggest the decisions that a hypothetical ideal observer (omniscient, imaginative, disinterested, dispassionate and consistent) who shares a value perspective with the individual, would adopt in certain particular situations.

With regard to the previous models, these two proposals would lead to an advancement in terms of autonomy since, in both cases, the involvement of the user is solicited in the determination of what is correct, conditioning the entire process to their particular values and final approval. But is the increase in autonomy achieved with this model significant? Can it be asserted that a moral enhancement in the user would truly occur? If we adopt the view that moral enhancement consists of increasing the competency to autonomously choose one's own decisions, I do not consider this third model to be suitable either. Once the subject has chosen the reference values required by the system, without the need for any reflection, their role is reduced to either accepting the result of the deliberation of the virtual advisor or not. And if it is ultimately accepted, their identification with the prescriptions recommended by the advisor will not be the result of a reflective process. The user can engage little in the reflection or deliberation on moral judgments if they arise entirely from the system and are based on a totally external process of determination. As it is not necessary to understand the rational connections between the values entered into the system and its conclusions, it is foreseeable that their moral abilities will not improve and that, without the help of the advisor, the person would continue making the same decisions as before, without any progress. Moreover, this interpretation of the relationship between the advisor and the user is hardly conducive to the user reconsidering their position. The user can indeed, at any time, change the personal values provided to the system, but it is unlikely they will do so. Savulescu and Maslen (2015, p. 92) recognise this when they assert that the use of their proposed enhancement system could encourage deference more than "deep reflection". As people are generally reluctant to change their moral values, it is foreseeable that they would be even more so if they believed that their decision was based on the advice of a supposedly reliable computer system (Lara & Deckers, 2020, p. 281).

In conclusion, the four moral enhancement models examined thus far —the biotechnological as well as the three based on AI— are unviable because they result in either a decrease or at least no increase in personal autonomy. Focusing on directly altering the moral behaviour of individuals, they neglect that this cannot be done unless a particular condition is met, namely that the behavioural change is a genuine process of self-determination. These models can thus only derive interventions or assistance more akin to mere behavioural control, rather than prepare the individual to make moral decisions. In short, it could be said that the models, despite having emerged with the intention to strengthen morality, are ultimately only able to override it.

## SocrAI, the Socratic Assistant

To overcome this deficiency in the autonomy of the previous models, in this section, I will formulate an alternative proposal of moral AIenhancement. It will consist of an expanded version of the virtual assistant that Jan Deckers and I devised in an article published in the journal *Neuroethics* (Lara & Deckers, 2020). It was inspired by the dialectical method adopted by Socrates in his dialogues, which aimed to help his interlocutors to reach definitions of concepts, usually of some virtue, on their own. The key difference between the Socratic approach and ours was that we used the method to promote moral learning. The interaction between the virtual assistant and the human user would be based on continuous questioning and aimed at developing the user’s capacities to evaluate and establish moral beliefs and values following requirements of empirical, conceptual, logical-argumentative and ethical rigour (Lara & Deckers, pp. 283–284).

### An Artificial Agent with a Hybrid Design

It is important to start by considering the technical characteristics that could make the assistant I propose here, which I will call SocrAI, a reality. It would be a conversational bot, in principle without a robotic "body". It could be categorised as a moral machine or an Artificial Moral Agent (AMA) and as such, it would be "capable of engaging in autonomous moral reasoning, that is, moral reasoning without the direct real-time input from a human user" (Wynsberghe & Robbins, 2019, p. 721). It would therefore meet the three essential criteria of an AMA: interactivity, autonomy and adaptability (Floridi & Sanders, 2004, pp. 357–358). SocrAI would have the capacity to respond to environmental inputs which, in this case, would be the user's answers to its questions (interactivity); it would itself make ethical judgements about the user's answers, in particular about whether or not they meet the aforementioned normative requirements of empirical, conceptual, logical-argumentative and ethical rigour (autonomy) and would act by applying these ethical judgements, without real-time human input, to formulating questions and suggestions to the complex and novel situations that users would pose with their different previous answers (adaptability). Still, it should be clear that we are not talking here about a full ethical bot, at the highest level of Moor's (2009) gradation of AMAs, with consciousness, intentionality and free will.^3^ Rather, SocrAI would be at Moor's previous level (Level 3), in the group of "explicit ethical agents", those bots that would use ethical categories as part of their programming, not simply to govern their behaviour according to specific guidelines, but to make it the result of an explicit representation of ethical principles (Anderson & Anderson, 2007, p. 15). AMAs at this level "have general principles or rules of ethical conduct that will be adjusted or interpreted to fit various kinds of situations" (Moor, 2009, p. 20). Another difference between these AMAs and those of the top level is that their scope is usually restricted, thus being governed by a "narrow artificial intelligence", which, unlike the "general" one, assumes a high degree of functionality within a limited scope (Bostrom, 2014). SocrAI would thus constitute an AMA whose ethical programming would ultimately serve to improve the moral education of users. This means that when designing it, in addition to the aforementioned normative requirements of good deliberation, requirements exclusive to an educational purpose would have to be taken into account, thus rendering the assistant ineffective for some other field.

Under the above characterisation of SocrAI as an AMA, the most promising way to design it would be according to a "hybrid" strategy, combining "top-down" principles and "bottom-up" learning (Wallach & Allen, 2009), albeit in a different way to that commonly used in current embedded ethics proposals for autonomous machines. In principle, the goal in our case is different: it is not about the machine doing the right thing, but about instructing the user so that he or she is better able to do it. Therefore, the instruction itself is not based on substantive ethical principles, but rather on general guidelines on how to reason better. In order to design SocrAI, therefore, it would be these guidelines (the normative requirements mentioned above) that would have to be codified in AI, so that they could be applied to specific cases, namely by evaluating the user's responses according to such guidelines. In principle, this would allow for an easier design of the assistant as it would avoid the main problem of the primary top-down proposals, which, based on substantive ethical principles, found it difficult to create algorithms that resolved the frequent conflicts between them. Even expertly agreed meta-principles would not be sufficient to resolve these conflicts (Wallach & Allen, 2009, pp. 84–97). However, if the normative criteria by which the virtual assistant is to be programmed will only be formal, of mere argumentative rigour, it is foreseeable that they will be consistent most of the time. The criterion of conceptual precision will seldom be at odds, for example, with argumentative logic or empirical support.

This search for a machine that does not seek to do the ethically correct thing in its operation or to advise the user in that respect also frees us from the problems most common to bottom-up proposals (about these problems, see Wallach & Allen, 2009, p. 110). Since the objective is only to solicit a better argument, there is no need to fear that the assistant, in learning its own strategies to optimise results, will end up, as would happen with other AMAs, doing or recommending what is in itself wrong, thus undoing or overriding built-in restraints. There would therefore be no problem if, for example, to get the user to be more conceptually precise, SocrAI learned that it would be better not to point out his inaccuracies, but to continue his arguments with them until the end. Nor would it be exposed to the dangers to the ethics of allowing the machine to learn what is right from a generalisation of specific cases. On the contrary, his learning from experience would be of great use to SocrAI, both to update the normative requirements so that they are more versatile for new user reactions and to increase its functional skills (data input, dialogic communication, argumentation, etc.). This should follow the lead of IBM's Project Debater, the first AI system that debates complex issues with humans and which would be an essential reference for the design of SocrAI. Project Debater configures its reasoning with data mining through supervised learning algorithms that analyse countless documents from legal and academic databases such as LexisNexis. The system collects well-structured arguments from these databases and extracts key phrases such as evidence for or against an assertion in order to construct its own argument (Slonim et al., 2021). Recently, the quality of the evidence that the system finds has improved considerably thanks to the adoption of BERT, the neural network for processing natural language created by Google. Thanks to the bidirectional (contextual) analysis of the words, it allows the search engine algorithms to better understand the user's language and respond more efficiently to their queries. But these achievements may be insignificant compared to those obtainable from the possible use of GPT-3, a powerful 175 billion parameter language generator developed by OpenAI. Unlike other models, it does not require pre-training on a large text corpus or fine-tuning to successfully perform a specific language task. GPT-3, by contrast, approaches the human ability to perform a whole range of tasks based on just a few instructions and examples (producing poetry, computer programming, music, jokes, articles and other results, frequently indistinguishable from human productions). The mining of arguments used by Project Debater is also being developed to be able to evaluate the quality of the arguments, for example, by detecting cognitive biases (Heaven, 2020).

However, it is important to qualify that, although Project Debater and GPT-3 are important techniques to consider for implementing the virtual assistant I am proposing, they will require significant adaptation to the purposes of this virtual assistant. Note that these techniques are aimed at achieving computational systems that argue in the most convincing way for a human user or listener. In our case, such rhetorical possibilities should be redirected towards the goals of our Socratic enhancement project.

Having outlined some guidelines for the design of SocrAI, in what follows I will argue that this virtual assistant could be the realisation of a moral enhancement model that not only respects but also increases moral autonomy. SocrAI would achieve this thanks to three traits that would differentiate it from the models presented thus far: educational guidance, full participation of the user and value neutrality.

### Educational Guidance and Full Participation of the User

Essential for the increase in autonomy, first, is the fact that the aim of this assistant is not to directly and immediately alter the behaviour of the person (as in the case of bioenhancement or the other AIenhancement proposals). The objective now would be for the user, with the exercise of their deliberative capacities, to learn to decide better and, with time, this would favour the ability to do so on one's own. Thanks to the inquisitive dialogue, the virtual assistant will make the person aware of their possible errors and they will feel motivated, where appropriate, either to respond as to why they believe they are not errors or to avoid them with revised positions.^4^ It is foreseeable that, with this dialectical training, the person will acquire the capacity to make decisions critically and self-sufficiently in the future.^5^

Second, SocrAI would strengthen the autonomy of the user because, thanks to this constant interaction, the user would be compelled to achieve a high degree of participation in the enhancement process. In the previous models it could be said that the involvement of the individual was either zero—the enhancement a result of either biological interventions or highly controlled computer systems—, or modest—limited to providing values and to either accepting the conclusive recommendation of the advisor or not. In all of them, it could be said that technology, in one way or another, decided for the individual. However, with SocrAI, the individual plays a dominant role in the decision-making and learning process, firstly, by providing a tentative solution to the moral questions that arise and, then, by responding to the inquisitive scrutiny of that solution by the machine, as Socrates did, by formulating questions and revealing flaws in the answers given by his interlocutor. Thanks to this interactive process, the user is compelled to reflect on their initial value positions and revise them where appropriate.

One might wonder to what extent the SocrAI user would want to participate in such a demanding interactive process in which he or she must be willing to respond to so many questions and suggestions from the computer, as well as to subsequently revise, where appropriate, postulates previously undisputed. I think the best way to get an idea of how collaborative the user's stance might be would be to look into the educational possibilities of the Socratic method. These possibilities depend very much on how we understand the method itself. If, as in the early Platonic dialogues, the aim is to make the interlocutor aware of his or her ignorance through the Socrates' own supposed ignorance, which, paradoxically, does not prevent Socrates from using a particular doctrine (as he does in the *Meno*), the resulting atmosphere can only be confrontational. In this case, "the process is generally not enjoyed by the interlocutors, and their reactions are often tense and hostile" (Brickhouse & Smith, 2009, p. 188). The attitude of the interlocutor will change, however, if, as is evident in the *Theaetetus*, the instructor makes it clear that he is not an expert in any doctrine or substantive knowledge, but only in a technique which, like that of the midwife, grants others the ability to "give birth" themselves to genuine wisdom that Socrates does not really have. This other understanding of the Socratic method may favour a more cooperative attitude on the part of the interlocutor in two ways: either because the latter feels like part of a collective enquiry in which everyone shares the love of learning in a group (Cicchino, 2001; Mintz, 2006; Strong, 1997), or because an educated person can perceive the sincere contribution of an instructor who does not intend to indoctrinate him, but only to favour his own personal development. In the latter case, one would value the work of the instructor in the same way as one values the care work of the midwife who, following the analogy, only intends to provide the best possible care. It would be valued because the questioning of one's own beliefs by the other is essentially productive (Brickhouse & Smith, 2009, p. 189). For the SocrAI user to participate in the enhancement process, the first way would not be valid, as such communal and affective links between the machine and the human would hardly exist. The second way, according to which SocrAI could be seen as a non-human assistant at the mere service of deliberative enhancement, would appear more promising. However, we should not naïvely rule out any user discouragement. The assistant's rebuttals and observations will confuse him or her and, in many cases, lead him or her to abandon what he or she previously held to be true (let us not forget that this is also the essential aim of Socrates). In many cases, this will not be pleasant for the user.^6^ Even so, the discouragement may be compensated by the advantages and satisfaction of an examined life. As a certain version of the Socratic method intended, doubts and the recognition of our inconsistencies as the sole causes of our ignorance might give more meaning to our experiences and circumstances (Brickhouse & Smith, 1994, pp. 17–18, 2009, p. 190; Haroutunian-Gordon, 1991, p. 14). An incentive to be wiser may be even more potent in the case of the virtual assistant user because the virtual assistant, unlike Socrates, does not believe that what we should be aiming for is an objective and universal truth. By SocrAI only expecting us to exercise certain deliberative capacities, but without presupposing substantive ethical principles, the fear of being led surreptitiously to some doctrine (as was the case with the early version of the dialogues) will be reduced, as will the trauma of having to abandon one's own principles (since it will always be easier to abandon them due to their being based on conceptual, empirical or even ethical inaccuracies than because they are contrary to a single true ethical theory). Still, I must acknowledge that, ultimately, willingness to participate in the process will be reserved for those who, to some extent, share the Socratic maxim that "an unexamined life is not worth living" (*Aporia*, 38th).

## Neutrality

There are therefore theoretical reasons to believe that the user of this technology, given certain conditions, would be motivated to actively participate in the constant interaction it would require. As we have seen, this interaction will be geared toward training the person for that personal and thoughtful adoption of values that characterises autonomy. But clearly, in no case can we claim that the values adopted after this participatory process are distinctly those of the person if the process was heavily directed by some value framework entered into the system by the designer. I therefore highlight, as a third attribute to boost autonomy as self-determination, the fact that SocrAI would be designed to guarantee the neutrality of the system concerning *substantive* values. Moreover, this emphasis on maximum personal freedom would be bolstered by SocrAI being designed from the perspective of a strong commitment to the *procedural* values of minimal and open rationality.

For the latter, I rely in part on the idea of procedural moral enhancement proposed by Schaefer and Savulescu (2019). Drawing on some ideas from J. Rawls' reflective equilibrium method, these authors identify some criteria that, without presupposing any substantive principles, could make people's judgements more morally reliable. The criteria outlined in Lara & Deckers (2020, pp. 283–284) for the content and the sequences of the SocrAI questions coincided with some of those proposed in Schaefer and Savulescu (2019), particularly those pertaining to logical competence, conceptual understanding and empirical rigour. We thus considered that it would be important for the assistant to improve the user's ability to, for example, argue according to logical rules or to detect fallacies in reasoning. We also proposed as functional criteria for SocrAI that, thanks to its extensive and rapid handling of big data, it should demand from the user fidelity to the facts and precision with regard to the concepts that are relevant in each moral judgement. When Schaefer and Savulescu (2019, p. 77) refer to the criterion of conceptual understanding, they include in this "a clear understanding of the content, strength and scope of moral ideas". This coincides with our requirement that the assistant should be designed to enrich the user's decision-making with knowledge of the positions of the main ethical theories regarding the issue in question.

Our procedural enhancement proposal differed from that of Schaefer & Savulescu, however, in that we added two more functional criteria. First, we introduced the monitoring of the user's physiology, mental states and environment, alerting the user of certain factors that could negatively affect his or her decision-making and, second, the functionality of the assistant to recommend how to implement decisions.

In the remainder of the section, I wish to reinforce the emphasis of SocrAI on procedural neutrality by doing two things. First, by adding a new functional criterion to those argued in Lara & Deckers (2020), thus enabling our decisions to be made from an empathetic perspective.^7^ Some may wonder whether this entails an attempt to direct the user toward certain substantive values such as it being fine to be concerned about the well-being of others. This would certainly be so if we were to understand empathy as the altruistic predisposition to feel like the other and, were this the case, to wish them not to suffer. But aside from “compassionate” (Batson, 2009; Batson et al., 2009; Darwall, 1998), empathy can also be “cognitive” (Fisher 2017, pp. 236–237; Seinfeld et al., 2018, p. 1; Bailenson, 2018, pp. 79–80). The latter is identified with a capacity to imagine how the other thinks and feels based on what he says or does and on the knowledge available regarding his character, values and desires. It would therefore consist of an emotionless capability to presume the subjective experience of someone occupying a different position, without entailing the desire to help them when the experience is painful. It is the demand for this type of cognitive empathy that could fit with a neutral and procedural proposal of moral enhancement like the one argued here.

This empathic capacity to determine how the other thinks and feels has traditionally been exercised in many ways: with extrapolations of profiles of like-minded people, mental experiments, psychological generalisations, etc. The aspiration common to these strategies is to overcome the limitations of our own imagination such as lack of relevant information, fatigue and biases. These limitations could be more easily surmounted, however, if our assistant drew on augmented and virtual reality technologies strongly linked to AI to facilitate cognitive empathy (Rueda & Lara, 2020). These technologies would provide the user with immersive experiences in computer-generated digital scenarios. By synchronising their real movements with those of the avatar in which they are embodied, the user could subjectively leave their physical reality and “be in” the projected virtual world (Shriram et al., 2017, p. 312; Slater and Sanchez-Vives 2016; Fenlhofer et al. 2015, p. 49; Won et al., 2015, p. 6; Seinfeld et al., 2018, p. 1). This would ensure a minimal imaginative effort required of the user to cognitively empathise with another since, to understand their perspective, the user would need only focus on the virtual experience (Banakou et al., 2016; Seinfeld et al., 2018, p. 7). A well-configured programme for this purpose could provide a high degree of realism given the rich sensorial nuances that these new technologies would transmit to the user (Ahn et al., 2013, p. 10) and the fidelity to the intended perspective (Ramirez & LaBarge, 2018). Furthermore, if certain precautions are taken, such as using avatars to embody roles and not particular personalities (Herrera et al., 2018; Loon et al., 2018), these technologies could come close to obtaining an exclusively cognitive empathy free of biases.^8^

This particular efficiency of SocrAI to make the user understand how others think and feel in the most authentic way possible is essential to the neutral autonomy required in the field of morality. This autonomy is achieved when the person is in a position to independently attain values that, whilst also their own, since they are *moral*, must to some extent be universal. In other words, the values must be justifiable with reasons formulated from this impersonal (neutral) perspective of equal consideration of the beliefs and interests of all formally required by the field of morality.

The second thing I wish to do here to support the emphasis of SocrAI on neutrality is to respond to the possible objection of whether this emphasis would lead to an ethical scepticism that would invalidate any attempt at moral enhancement. How can we say that progress has been made without a substantive value with which to evaluate the change? Absent this value, the enhancement would be reduced —it would be objected— to a greater capacity for argument, regardless of the conclusion that may be reached. The result would then be the formation of an empty and false person through a virtual assistant closer to the sophists than the Socrates that we proposed as a reference.

However, in my opinion, this objection rests on an unfounded distrust in the normative achievements of a procedural ethic like the one underpinning my proposal. By just requiring, as this ethic does, that the judgments we assert be consistent, conceptually precise and empirically founded, many of the most widely accepted moral positions would have to be rejected. Not just any substantive position would therefore suffice and those that pass these kinds of ethics tests could only be reflected in demanding and highly specific prescriptions.

That said, it must be qualified that although value scepticism is not an inevitable consequence of our proposal, pluralism would be. We cannot forgo the premise that there can be several acceptable and irreconcilable value alternatives if we think that autonomy is characteristic of morality. Moreover, value plurality would also represent a good tool precisely for increasing that same autonomy. It will always be easier for the individual to critically determine their own moral judgments if the assistant presents, in a neutral manner, the widest range of procedurally valid ethical options possible.^9^ The Socratic appeal to personal inquiry through dialogue is therefore crucial to being autonomous; but so is the sophist reminder that what is morally valid does not always concur.

## SocrAI *Versus* the Socratic Teacher

The conclusion that we can draw from the above is that there are reasons to believe that if we wish to make use of technology to morally enhance individuals, SocrAI could be the ideal choice. Thanks to a dialectical method based on neutrality and deliberative rigour, this virtual assistant would strengthen the capacities necessary for making truly moral (autonomous) decisions. But it makes perfect sense to then wonder whether it is necessary to make use of technology to achieve this. Could we not do the same thing with human instructors who, in the style of Socrates himself, were to follow the same method as SocrAI? They would be philosophically and ethically trained instructors, with good oratorical skills, with access to all of the information available in computerised databases and who would converse with their pupils, from a point of neutrality, with the aim of better deliberation. To respond to this challenge, in this section I will compare those two hypothetical assistants, the virtual and the human, by virtue of their supposed advantages in terms of moral enhancement. I will focus on three aspects that I consider essential to the comparison: teaching skills, value neutrality and power to motivate.

### Teaching Skills

To evaluate the efficiency of the educational function of both assistants, I will adhere to three criteria that I consider important: their information supply and management capacity, their agility in dialogue and their availability.

First, to satisfy the functional criteria of our procedural proposal (conceptual precision, empirical support, logical demands, etc.) both assistants should bolster their questions and suggestions to the individual with information on science, linguistics, logic, argumentation theory, etc. It seems clear that although the human instructor could access the same databases as SocrAI to obtain this information, the latter, thanks to its AI resources, could process this information more quickly, tirelessly and in accordance with a greater number of parameters.

But the relevant information for the enhancement of decision-making would not come from databases alone. To this end, it would also be important for the individual to know at all times whether the conditions are suitable to decide. In this, technology could also be much more efficient. By monitoring the user and their environment, an assistant like SocrAI could obtain and utilise information regarding the existence of suitable mental and environmental conditions for deliberation faster and more efficiently than the human assistant. These include sufficient sleep, little time between meals, a lack of fatigue, absence of neuronal alterations, a lack of excessive heat and sound in the environment, etc. (Savulescu & Maslen 2015, pp. 85–86).

The virtual assistant would therefore be preferable with regard to the speed in obtaining and processing a large amount of information from databases and monitoring that proves relevant for improving decisions and, in the long-term, the capacities to make them autonomously. But this greater speed cannot be extended to all of the areas involved in the process, for example, the dialogue with the user. Different versions of Natural Language Processing are used to “converse” with virtual assistants which, due to their deficiencies in detecting many nuances and implicitly understood elements of human language, turn the dialogue with virtual assistants into something very slow and, at times, ineffective.^10^ Only time will tell if it will be possible to technically overcome such communicative deficiencies and the virtual assistant will reach, in this respect, the level of a human instructor, currently much more agile in dialogue.^11^

A third criterion for comparing the educational potential of SocrAI and its human opponent with regard to moral enhancement would be the degree to which both would be available. It appears that here, in principle, the former would be worse. It is anticipated that given its technical sophistication and consequent high cost, it would be beyond the reach of many of its potential users. Nevertheless, our experience with the marketing of other advanced technology products, such as mobile phones or computers, would justify the belief in a likely price reduction, over time, of virtual assistants and in their corresponding availability to the general public. This foreseeable process could even be accelerated if public institutions, aware of the social benefits of this type of assistant, invested in its development, made it available to underprivileged citizens through subsidies, or included it in the list of social services they offer to citizens.

Furthermore, if an assistant like SocrAI becomes commercially accessible, via price reduction or public subsidy, it would be more widely available than its human counterpart for the mere fact that machines can be used whenever the user desires, and not only on the days and times established for the necessarily regulated services of human instructors given their inherent professional and biological limitations. It should also be taken into account that, in contrast to the universal availability of a SocrAI thanks to multilingual translation applications, which are already quite advanced and easily used by the assistant, we would have human instructors who, due to their limitations in the learning of new languages, would have to be trained for different geolinguistic areas.

### Neutrality

As we have seen, neutrality is important to the moral enhancement of individuals because it protects the process from potential attacks on personal autonomy. To that end, we proposed that the interaction between SocrAI and the individual be governed by strictly formal and procedural criteria, and thus detached from any bias that could excessively or surreptitiously influence them and thus limit the free and reflective pursuit of one's own values.

It seems that this criterion of neutrality could be better satisfied by machines which, in principle—provided that they are not manipulated to do otherwise—, would be free from the biased emotions and attitudes that evolutionarily characterise human beings (Persson & Savulescu, 2012). But this initial lack of emotions inherent in machines does not impede certain factors involved in their design, even when not knowingly biased, to impact the emotions of human users and compromise that neutrality and autonomy that would allow for their moral advancement. The following precautions should be taken into account so that this does not occur.

First, the virtual assistant should be designed is such a way that the objective of its interaction with the user is limited, as my proposal advocates, to the better exercise of strictly intellectual (cognitive and deliberative) capacities.

Second, to prevent—or to reduce as much as possible—the virtual assistant from generating emotions in humans that pervert their open value development, it should be designed without any discernible human or animal form. Recent studies show that companion robots, manufactured with the appearance of pets or human beings, elicit in the users consolidated emotions of attachment to the robots which even lead to attributing some type of mental state or social status to them (Friedman et al., 2003; Melson et al., 2009). Therefore, if a non-provocative design is used, the user would be emotionally distanced from the assistant, facilitating reflective independence.

With that same intention of optimally reducing emotional influences, we should expressly forgo the “affective computing” techniques with which automated systems aim to imitate user emotions and attitudes. Based on the psychological tendency for people of a similar nature to be attracted to each other, companion robots emotionally identical to the user are designed with these techniques to gain their trust and thus fulfil their emotional deficits or make them change their unhealthy habits. In our case, it is clear that interaction based on this emotional affinity could lead to either an excessive dependence of the user on the assistant or easier manipulation of them by a malicious designer. In both cases, the results are counterproductive to a virtual assistant that only seeks the development of intellectual abilities, with maximum autonomy.

It could be objected that the necessary lack of emotions in the relationship between the assistant and its user could detract from its effectiveness as, by making the assistant so cold, the user might experience a certain discomfort or demotivation. This would contrast—the objection would add—with an instruction carried out by a human with whom the relationship would never be as cold and which would free us from the strange sensation of performing an activity that is usually done between humans, debating or training, with the machine.

Even so, there are reasons to believe that this understandable perplexity in light of such a novel (conversational and formative) relationship with cold machines could gradually disappear. In fact, this has occurred in the past whenever, due to the advent of new technologies, we have begun to perform activities with machines that we previously did only with humans, such as talking on the telephone or shopping online. It should be added that this unemotional relationship between human and machine could even, in certain situations, be more efficient for moral enhancement. This would occur, for example, when the users are people who, due to their violent (Klincewicz, 2019, p. 443), irritable or shy nature have difficulties interacting with a human instructor.

### Motivation

Given that the aim is to devise an assistant for moral enhancement, it is obvious that it must not function solely for the user to be aware of the deficiencies in their decisions and to know how to avoid them. It must also be useful so that, in practice, these new skills will cause them to alter their values and behaviour. First, neither of our two assistants would be very good at this, as both aim to influence only the deliberative and rational aspects of the person, but not the motivational. I have even argued that the design of the virtual assistant, in order to preserve autonomy and neutrality, should be particularly careful not to directly influence the user's emotions, which are the quintessential source of motivation.

This notwithstanding, I believe that both assistants could overcome this motivational deficit without having to thereby abandon their common aim of exclusively intellectual enhancement. I therefore rely on what we can call *the persuasive power of reason*. Whilst it may be true that an argument on its own is not motivational because it is formally differentiable from desires and emotions, which are the quintessential engines for action, these desires and emotions can also be triggered by a strongly convincing argument. In this sense, it can then be argued that both assistants for argumentative deliberation could at least be *indirectly* motivational.

Furthermore, it makes sense to expect that, in reality, our two assistants, even though only concerned with intellectual matters, would be highly effective for attitudinal change in the recipient of the instruction. For this expectation, we could rely on the plausible assumption that the arguments are ultimately more motivating when, in addition to being convincing, they are the result of personal effort. It follows from our previous remarks on the Socratic method that both SocrAI and our Socratic teacher would invite participation because they would follow a version of this method that does not aim to direct them according to a predetermined substantive framework of values. The objective is to help them to decide on their own and with strict neutrality. The individual will therefore always perceive the decisions resulting from the dialogue with the virtual assistant, or with the human instructor, as *their own* and this will make them much more motivating. Moreover, the motivational force of the decisions will increase even more as the individual considers that such decisions are the result of a demanding learning process in which it was constantly necessary to debate with an expert.

Both assistants could thus become more motivating than they might have originally seemed. But, would one of them be preferable in this regard? There are reasons to believe that SocrAI would be preferable, especially due to that persuasive force derivable from the positive valuation that the user would make of the deliberative process. In my opinion, people would appreciate the arguments more when they come from a dialogue with a virtual assistant because the observations it makes, provided that certain precautions are taken, could seem more reliable than those of the human instructor. This assertion would make sense according to the two main dimensions from which trust is understood (Roff & Danks, 2018). On the one hand, there is the trust we normally place in machines and artefacts, which is largely a matter of predictability and reliability. In line with this, there are studies that suggest that the degree of trustworthiness generated in us by computerised and automated systems really depends on the effectiveness that we expect from them. This expectation of effectiveness stems from our beliefs about how many problems we consider them to have been able to resolve in the past, and how many we anticipate they will resolve in future situations (Carlson et al., 2014, p. 4). If we use these criteria to compare the trust that our two assistants would generate, it is foreseeable that the virtual one would be evaluated more positively, given the widespread belief that machines lack many of the cognitive and volitional limitations characteristic of humans (Muir, 1987; Klincewizc, 2016, p. 181). As such, SocrAI could boost its (indirect) motivating force—and by far surpass the human Socrates—if it were designed to provide convincing evidence of its effectiveness. In this case, this would not be achieved by showing its success rate or making its decisions more understandable, as some authors recommend for automated systems in general (Lee & See, 2004), but rather by allowing the user to pause the dialogue at any time in order to demand that the assistant explain the origin and authority of the source of the data being used in its questions and suggestions.

But, on the other hand, there is the much more complicated form of trust, more characteristic of interpersonal relationships, which depends mainly on understanding rather than predicting the other's behaviour. It is necessary to understand the underlying values, preferences and beliefs that present a reason for his or her course of action. Regarding this second dimension of trust, in our case, the expectations that SocrAI or the human instructor arouse in us will depend very much on how we conceive the ontology of the virtual assistant. Given the limited degree of autonomous learning and its lack of other more complex aspects, such as consciousness, SocrAI’s behaviour will leave less room for misunderstanding and the expectations generated will always be stronger. On the contrary, the expectations of the Socratic instructor's disciple will always remain at the mercy of an unexpected response, understandable by the much more autonomous and emotional nature of humans.

Another aspect of SocrAI that would present it as more motivating than its human counterpart has to do with the potential for its use of VR technology in increasing the cognitive empathy referred to earlier. We should not forget that the problem here is how to translate the decisions reached into a willingness of the individual to act accordingly and, as they are based on deliberations of morality, that they must be based on reasons adopted from the impersonal point of view that characterises this normative field. SocrAI would do this better because, by allowing the user to virtually embody the role of other involved subjects, their perspectives could be more faithfully and vividly understood and the user would thus feel more inclined to take them seriously and act impersonally.

## Conclusion

The key in moral education is that it be pursued while respecting and promoting personal autonomy. Educators should avoid the mistake of limiting the capacities of individuals to freely and reflectively determine their own values by attempting to enhance their behaviour directly. On the contrary, they must do what they can to ensure that those being educated, at least at an advanced age, actively participate in this process in order to assume the values that will define them and give meaning to their lives. The problem with current proposals for moral enhancement through new technologies is that they treat the subject of their interventions as a "passive recipient". Moral bioenhancement does so because it aims to change the motivation of the individual by bypassing the reflection and gradual assimilation of values that should accompany any adoption of new identity traits. This constitutes a passivity that would also occur in proposals for moral AIenhancement based on ethical machines that either replace humans in decision-making, or surreptitiously direct them to do the right thing, or simply advise them based on their own supposedly undisputed values.

In this article, I have developed and justified a new moral AIenhancement model focused on autonomy. It involves a virtual assistant that, rather than making moral decisions for us, instructs us, through dialogue, so that we make them ourselves by following criteria of neutrality and deliberative rigour. I have also argued that although, in principle, this could be achieved through a human instructor using a similar method of instruction, it would be significantly improved with the proposed virtual assistant, provided that progress in its communicative capacity is made, that people can acquire access to it, and that particular precautions are taken in its design so that, for example, it does not directly influence the user's emotions and the sources of its observations are transparent, thus generating maximum confidence.

## Footnotes

1 Although this has been the dominant trend, there have also been authors who have questioned the moral effectiveness of focusing on motivational aspects and have argued instead for the desirability of increasing by biotechnological means either the moral agent's deliberative domain (Harris 2016) or both the cognitive and the emotional (DeGrazia 2014).

2 Dietrich (2001, p. 531) is so pessimistic about the moral nature of human beings, and so optimistic about the possibilities of AI, that he advocates an obligation to "usher in our own extinction" in order to create a better world inhabited solely by ethical robots. These robots, which he calls “Homo sapiens 2.0”, would be improved versions of ourselves because they would have achieved that "Copernican turn" —unattainable to us due to our biological conditioning— which allows one to act from the belief that one is not the centre of the universe (Dietrich 2001, pp. 532–533, 536).

3 There is much debate about whether it will ever be possible to have a conscious machine (Peterson 2012; Torrance 2008; Wallach 2010) and whether consciousness, intentionality and free will are inescapable features of full moral agency (Floridi & Sanders 2004; Gunkel 2014; Himma 2009; Sparrow 2012).

4 To concretise the idea of moral progress that should inspire SocrAI's educational pretension, the Stoics' conception of the sage, a figure from whom these philosophers derived their practical advice for a virtuous and happy life, would also be very useful. A sage whom, by the way, they identified with Socrates. Thus, in line with the Socratic method's claims to self-reflection and coherence, SocrAI should be seen to acquire the Stoic skills of pointing out to the user how some of his mistakes depend on a blind and quick acceptance of emotions, irrational fears (about matters beyond our control) and an excessively materialistic and self-centred outlook. The Stoics were convinced that perceiving the sources of these types of errors was an essential element of an "examined life", as claimed by Socrates. On the practical advice of the Stoics and the influence on them of the figure of Socrates and his method, see, for example, Pigliucci (2017, pp. 201–221); Adamson (2015, pp. 73–100); Brown (2006).

5 The dialectical process could be accompanied by other types of activities that share this educational purpose. Due to their virtual condition and their derivation from AI, particularly relevant here would be "serious games", video games designed more for learning than for entertainment (Abt 1987, p. 9). Thus, Staines et al. (2019) propose Morality Play, a model of moral expertise game with which to improve skills in different functional areas of morality. Video games of this type would allow the user to position him/herself for different virtual scenarios in which to apply and refine progress in the intellectual skills sought by SocrAI.

6 This predictably different response to the Socratic method is corroborated by the fact that in the Platonic dialogues not all the interviewees are enthusiastic about Socrates' questions. Euthyphro is puzzled and abruptly ends the dialogue by postponing it for another time when he is not in such a hurry (*Euthyphro*, 15e). So does Protagoras (*Protagoras*, 361e). The provision of sufficient time for debate may be an important factor in ensuring that the embarrassment produced by the method translates into motivation for the interviewee, but his attention span, cultural level, argumentative practice or intellectual curiosity are likely to be important as well. How the interviewer poses the questions and the degree to which he or she uses the method will also matter, of course. For this purpose, published works on pedagogical experiences with the Socratic method can be very useful. See, for example, McAllister (2018).

7 This criterion is also included, albeit to a lesser extent, in Schaefer & Savulescu (2019, pp. 79–80)'s procedural proposal. In the same vein, Paulo (2018) advocates a “moral-epistemic enhancement” that would justify certain use of biomedical interventions that facilitate the capacity to adopt the perspective of another.

8 The risks, which for the proposal defended here would involve not taking these kinds of precautions, are made clear in the current success of NGOs and institutions in using VR to raise public awareness regarding certain situations that breed suffering in animals and humans (Fisher 2017, pp. 233–236). They thus increase a compassionate empathy that, whilst justified in utilitarian terms, is partial and can easily be manipulated (Prinz 2011; Masto 2015, p. 76; Bloom 2016), often jeopardising the autonomy of the user.

9 This ethical pluralism contrasts with most of the current major systems for assistance in moral decision-making which always take the side of a particular ethical theory: the utilitarian JEREMY, the Rawlsian-Rossian MedEthEx or the casuistic Truth-Teller and SIROCCO (Lara & Deckers, 2020, p. 282). However, precisely due to these preferences, none of these systems can be universally acceptable. SocrAI, by contrast, would boast the advantage that, due to its ethical pluralism, it could be taken on by academics and users of a different ethical orientation.

10 See, for example, the contrast between the argumentative efficiency and the slowness in the replies expressed by IBM's Project Debater in its encounter in a debate competition with Naris Natarajan, a champion in this type of competition: https://www.youtube.com/watch?v=3_yy0dnIc58.

11 The achievements of the recent GPT-3, the great breakthrough in language generating systems—referred to above in the text—may be decisive in bridging this gap.
